# Triplet therapy with afatinib, cetuximab, and bevacizumab induces deep remission in lung cancer cells harboring EGFR T790M *in vivo*

**DOI:** 10.1002/1878-0261.12063

**Published:** 2017-05-02

**Authors:** Kenichiro Kudo, Kadoaki Ohashi, Go Makimoto, Hisao Higo, Yuka Kato, Hiroe Kayatani, Yasuko Kurata, Yoichiro Takami, Daisuke Minami, Takashi Ninomiya, Toshio Kubo, Eiki Ichihara, Akiko Sato, Katsuyuki Hotta, Tadashi Yoshino, Mitsune Tanimoto, Katsuyuki Kiura

**Affiliations:** ^1^ Department of Hematology, Oncology and Respiratory Medicine Okayama University Graduate School of Medicine, Dentistry and Pharmaceutical Sciences Japan; ^2^ Department of Respiratory Medicine Okayama University Hospital Japan; ^3^ Department of Pharmacy Okayama University Hospital Japan; ^4^ Pharmaceutical Care and Health Sciences School of Pharmacy Shujitsu University Okayama Japan; ^5^ Center for Clinical Oncology Okayama University Hospital Japan; ^6^ Center for Innovative Clinical Medicine Okayama University Hospital Japan; ^7^ Department of Pathology Okayama University Graduate School of Medicine, Dentistry and Pharmaceutical Sciences Japan

**Keywords:** afatinib, bevacizumab, cetuximab, deep remission, EGFR T790M

## Abstract

Epidermal growth factor receptor (EGFR) tyrosine kinase inhibitors (TKIs) have changed the treatment strategy for EGFR‐mutant lung cancers; however, resistance usually occurs due to a secondary mutation, T790M, in EGFR. Combination therapy using afatinib and cetuximab has had good results in lung tumors harboring EGFR ^T790M^ mutations in clinical trials. The effect of bevacizumab, an antivascular endothelial growth factor (VEGF) antibody, combined with EGFR‐TKIs has also been investigated. We hypothesized that the dose of afatinib and cetuximab could be reduced by combination with bevacizumab and that the triplet therapy may result in better tumor inhibition with tolerable toxicity. Using a xenograft mouse model with H1975‐harboring EGFR^L^ ^858R+T790M^ and RPC‐9‐harboring EGFR ^19DEL+T790M^, we tested the efficacy of the triplet therapy with a modified dose of afatinib, cetuximab, and bevacizumab, and compared this therapy to single and double therapies. Triplet therapy with afatinib, cetuximab, and bevacizumab induced pathological complete remission in xenograft tumors with H1975 and RPC‐9 cells versus tumors treated with single or double therapies. We saw no body weight loss in the mice. The triple therapy induced a significant reduction in CD31‐positive vascular endothelial cells and increased cleaved caspase‐3‐positive cells in the tumors. This suggests that one mechanism underlying the deep remission could be suppression of neovascularization and induction of apoptosis by intensive inhibition of driver oncoproteins and VEGF. These results highlight the potential of afatinib, cetuximab, and bevacizumab to induce deep remission in tumors harboring EGFR^T^ ^790M^ mutations. Therefore, clinical trials of this combination therapy are warranted.

**Abbreviations**
EGFR: epidermal growth factor receptor
LC‐MS/MS: liquid chromatography–tandem mass spectrometry
MAPK: mitogen‐activated protein kinase
NSCLC: non‐small‐cell lung cancer
TKIs: tyrosine kinase inhibitors
VEGF: vascular endothelial growth factor

## 1 Introduction

The discovery of oncogenic driver mutations, including epidermal growth factor receptor (EGFR) mutations, has dramatically changed our practical strategy for patients with advanced non‐small‐cell lung cancer (NSCLC; Saito *et al*., 2016). Notably, patients with driver mutations that received matched targeted agents lived much longer than those with a driver and no targeted therapy (Kris *et al*., 2014).

Several randomized clinical trials have shown that first‐generation EGFR tyrosine kinase inhibitors (TKIs), including gefitinib and erlotinib, and second‐generation EGFR‐TKIs, including afatinib, achieved a good clinical response in lung cancers harboring activating *EGFR* mutations (Maemondo *et al*., 2010; Yang *et al*., 2015; Zhou *et al*., 2011). Of note, afatinib resulted in prolonged survival compared with platinum doublet chemotherapy, the first such improvement in 30 years (Yang *et al*., 2015). However, the majority of lung tumors acquire resistance to EGFR‐TKI, usually after about 12 months of treatment (Ohashi *et al*., 2013). Up to 60% of the lung tumors develop a secondary resistant mutation, T790M*,* in exon 20 of EGFR (Ohashi *et al*., 2012; Sequist *et al*., 2011).

Recently, a third‐generation EGFR‐TKI (osimertinib) that inhibits EGFR T790M has been approved in several countries, including the USA and Japan. Unfortunately, resistance to new‐generation EGFR‐TKIs is inevitable (Jänne *et al*., 2015; Planchard *et al*., 2015; Thress *et al*., 2015). As an alternative strategy, intensive EGFR inhibition combined with afatinib and a monoclonal anti‐EGFR antibody, cetuximab, showed a promising response in resistant tumors with EGFR T790M mutations in a phase Ib clinical trial (Janjigian *et al*., 2014; Regales *et al*., 2009). A randomized phase II/III trial is currently being conducted (NCT02438722); however, considering the results of the phase Ib trial, the effect of this drug combination still seems to be transient (Janjigian *et al*., 2014; Pirazzoli *et al*., 2014).

Antiangiogenic agents are thought to have several effects on tumors, including normalization of microvasculature and improvement of drug delivery (Ferrara and Kerbel, 2005; Jain, 2013). To further improve the EGFR‐TKI management strategy for lung tumors harboring EGFR mutations, we established a combination therapy that includes EGFR‐TKIs and an antivascular endothelial growth factor (VEGF) antibody, bevacizumab, to treat lung cancer tumors harboring EGFR T790M mutations *in vivo* (Ichihara *et al*., 2009; Ninomiya *et al*., 2013). Subsequently, the effect of combination therapy was assessed in treatment‐naïve patients in clinical trials. In a randomized phase II trial, the erlotinib and bevacizumab combination significantly prolonged progression‐free survival compared to erlotinib alone in patients with lung cancers harboring EGFR mutations (Seto *et al*., 2014). We also reported the promising effect of combination therapy using gefitinib and bevacizumab (Ichihara *et al*., 2015), and we are currently conducting a phase I/II clinical trial on the combination of afatinib and bevacizumab in the same population (UMIN000015944).

Taken together, we hypothesized that the addition of bevacizumab to the afatinib and cetuximab combination may allow for modification of the dose for each of the agents, resulting in better tumor inhibition and more tolerable toxicity. In this study, we showed that the triplet therapy, which included afatinib, cetuximab, and bevacizumab, induced pathological complete remission (CR) repeatedly in lung cancer cells harboring *EGFR T790M* mutations *in vivo*.

## 2 Materials and methods

### 2.1 Cell culture and growth inhibition *in vitro*

Gefitinib‐resistant adenocarcinoma cells (RPC‐9) harboring EGFR exon 19 deletion mutations (E746‐A750) and exon 20 missense mutations (T790M) were established in our laboratory (Ogino *et al*., 2007). The H1975 pulmonary adenocarcinoma cells carrying exon 21 missense mutations (L858R) and T790M cells were purchased from the American Type Culture Collection (Rockville, MD, USA). Cells were cultured at 37 °C with 5% CO_2_ in RPMI 1640 medium supplemented with 10% heat‐inactivated fetal bovine serum. Growth inhibition was determined using a modified 3‐(4,5‐dimethylthiazol‐2‐yl)‐2,5‐diphenyltetrazolium bromide (MTT) assay as described previously (Ogino *et al*., 2007). Each assay was performed in triplicate.

### 2.2 Reagents and antibodies

Afatinib was kindly provided by Boehringer‐Ingelheim (Ingelheim am Rhein, Germany). Gefitinib, cetuximab, and bevacizumab were purchased from EVELETH (Eveleth, MN, USA). Osimertinib was purchased from Selleck Chemicals (Houston, TX, USA). Rabbit antisera against EGFR, phospho‐specific (p) EGFR (pY1068), mitogen‐activated protein kinase (MAPK), pMAPK (pT202/pY204), Akt, pAkt (pSer473), cleaved caspase‐3, BIM, and GAPDH were purchased from Cell Signaling Technology (Danvers, MA, USA). Anti‐Ki‐67 antibody was purchased from Santa Cruz Biotechnology (Santa Cruz, CA, USA). Anti‐CD31 antibody was purchased from DIANOVA (Hamburg, Germany).

### 2.3 Immunoblotting analysis

Cells and frozen tissues were lysed in a radioimmunoprecipitation assay buffer [1% Triton X‐100, 0.1% SDS, 50 mmol·L^−1^ Tris/HCl (pH 7.4), 150 mmol·L^−1^ NaCl, 1 mmol·L^−1^ EDTA, 1 mmol·L^−1^ EGTA, 10 mmol·L^−1^ β‐glycerol phosphate, 10 mmol·L^−1^ NaF, 1 mmol·L^−1^ sodium orthovanadate‐containing protease inhibitor tablets (Roche Applied Sciences, Mannheim, Germany)]. Proteins were separated by electrophoresis on polyacrylamide gels, transferred onto nitrocellulose membranes, and probed with specific antibodies followed by detection with Enhanced Chemiluminescence Plus (GE Healthcare Biosciences, Piscataway, NJ, USA).

### 2.4 Xenograft model

Female athymic mice aged 5–7 weeks were purchased from Charles River Laboratories Japan (Yokohama, Japan). All mice were provided with sterilized food and water and housed in a barrier facility under a 12‐hour light/12‐hour dark cycle. Cells (2 × 10^6^) were injected bilaterally into the backs of the mice. Ten days after injection, the mice were randomly assigned to a group and were then treated with either monotherapy, double therapies, triplet therapy (4–10 mice per group), vehicle (p.o. five times a week), afatinib (p.o. 10 mg·kg^−1^, five times a week or 25 mg·kg^−1^, five times a week), cetuximab (i.p. 0.1 mg per mouse, once a week or 1 mg per mouse, twice a week), bevacizumab (i.p. 2 mg·kg^−1^, twice a week or 5 mg·kg^−1^, twice a week), or osimertinib (p.o. 5 mg·kg^−1^, five times a week). Tumor volume (width^2 ^× length/2) was determined twice a week. The administration period for each drug was 28 days, and the follow‐up time was a further 28 days.

### 2.5 Immunohistochemistry

Formalin‐fixed, paraffin‐embedded tissue blocks from the samples were cut to a thickness of 5 μm, placed on glass slides, and deparaffinized in xylene and graded alcohol for 10 min. The antigen was incubated in 10 mmol·L^−1^ sodium citrate buffer, pH 6.0, for 10 min in a 95 °C water bath. The sections were then blocked for endogenous peroxidase with 0.3% hydrogen peroxide in methanol. The slides were rinsed with TBS containing 0.1% Tween 20, and the sections were blocked with goat serum for 60 min. The sections were incubated with an anti‐EGFR monoclonal antibody, cleaved caspase‐3 antibody, Ki‐67 antibody, or anti‐mouse CD31 antibody overnight at 4 °C. Then, the sections were amplified using biotinylated anti‐rabbit antibodies and avidin–biotinylated horseradish peroxidase conjugate for 10 min (LSABTM2 Kit; DakoCytomation, Glostrup, Denmark), and reacted with 3,3‐diaminobenzidine. Finally, the sections were counterstained with hematoxylin. Cleaved caspase‐3‐ and Ki‐67‐positive cells, and CD31 blood vessels, were counted in five random fields (× 100).

### 2.6 Liquid chromatography–tandem mass spectrometry (LC‐MS/MS) conditions for afatinib quantification

Chromatographic separation was performed using a high‐performance liquid chromatography system (Agilent 1100 series; Agilent, Santa Clara, CA, USA) and a CAPCELL PAK C18 MGIII S‐5 (100 mm × 2.0 mm; i.d., 3 μm; SHISEIDO, Tokyo, Japan) analytical column at 40 °C. The isocratic mobile phase consisted of mobile phase A (0.1% formic acid) and mobile phase B (methanol; 40 : 60, v/v) at a flow rate of 0.3 mL·min^−1^. The solution was filtered using a 0.22‐μm membrane.

Mass spectrometric detection was performed on an AB SCIEX API 2000 triple quadrupole mass spectrometer (AB SCIEX, Toronto, ON, Canada). Data acquisition was performed using Analyst™ 1.6.1 software (AB SCIEX). The mass spectrometer was operated in positive ion mode. Optimized instrument settings were as follows: curtain gas, 10 psi; ion source gas 1, 30 psi; ion source gas 2, 50 psi; ion spray voltage, 4000 V; and turbo heater temperature, 500 °C. Quantification was performed in multiple reaction monitoring mode with mass‐to‐charge (*m*/*z*) transitions at 486.5 > 371.5 for afatinib.

### 2.7 Preparation of the standard solution

Stock solutions of standard afatinib were prepared at 1 mg·mL^−1^ in methanol. The stock solutions of CP were diluted to 20, 100, 200, 1000, and 2000 ng·mL^−1^ in water.

Xenografts collected from five to seven mice were weighed (10–30 mg) and homogenized in 200 μL of water. A mixture of 600 μL of acetonitrile with 0.5% acetic acid and 50 μL of standard afatinib solution was added to 200 μL of xenograft homogenate. The mixture was vortexed for 1 min, kept on ice for 10 min, then centrifuged at 10 390 ***g*** for 10 min. An 800 μL aliquot of the supernatant was transferred to a clean microtube and evaporated to dryness under vacuum at 65 °C for approximately 1 h. The dry extracts were reconstituted in 100 μL of a mixture of 0.1% formic acid/methanol (20 : 80, v/v) by vortex mixing for 30 s. The samples were centrifuged once more for 10 min at 10 390 ***g***, and 40 μL of the clear supernatant was injected into the HPLC system. All data are shown as amounts of afatinib per wet weight of collected xenograft.

### 2.8 Statistical analysis

Statistical analysis was performed using SPSS (SPSS, Chicago, IL, USA). Group differences were compared using Student's *t*‐test. A *P* value < 0.05 was considered statistically significant.

## 3 Results

### 3.1 The transient effect of afatinib plus cetuximab, or afatinib plus bevacizumab, in RPC‐9 xenograft models

**Figure 1:**
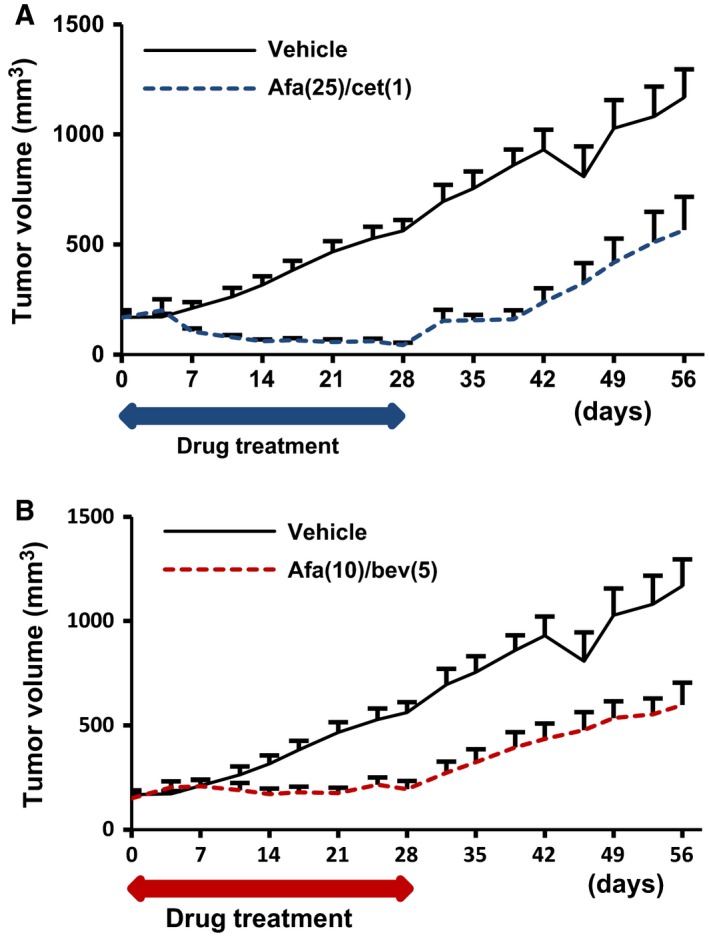
Transient effect of afatinib plus cetuximab or afatinib plus bevacizumab in xenograft tumors with RPC‐9 cells harboring epidermal growth factor receptor (EGFR) exon 19Del + T790M mutations. Afa, afatinib; Cet, cetuximab; Bev, bevacizumab. (A) The mice were treated with vehicle or afatinib (25 mg·kg^−1^, five times per week p.o.) plus cetuximab (1 mg per mouse, twice a week i.p.). Bars, SE. (B) The mice were treated with afatinib (10 mg·kg^−1^, five times per week p.o.) plus bevacizumab (5 mg·kg^−1^, twice a week i.p.). Bars, SE.

We first assessed the magnitude of remission induced by doublet therapies (afatinib plus cetuximab or afatinib plus bevacizumab) in RPC‐9 xenograft tumors harboring EGFR exon 19Del and T790M mutations (Ogino *et al*., 2007). A previous preclinical study demonstrated that the combination of afatinib (25 mg·kg^−1^, 5 days per week) with cetuximab (1 mg per mouse, twice a week) was more effective than each therapy alone in cells with T790M mutations (Regales *et al*., 2009). The same dose of afatinib plus cetuximab was administrated in a xenograft model using RPC‐9 cells for 4 weeks, and the mice were subsequently observed for 4 weeks after treatment cessation. As expected, the combination therapy significantly inhibited the xenograft tumors. Notably, prompt regrowth of tumors was confirmed after treatment discontinuation (Fig. 1A), and a trend toward body weight loss was observed in mice treated with combination therapies (Fig. S1A).

The magnitude of remission induced by afatinib (10 mg·kg^−1^, 5 days per week) and bevacizumab (5 mg·kg^−1^, 2 days per week) (Ninomiya *et al*., 2013) combination therapy was also assessed for 4 weeks during treatment, and a subsequent 4 weeks after treatment cessation. Again, the combination therapy significantly inhibited xenograft tumor growth for RPC‐9 cells, but the tumors quickly regrew during the observation period (Fig. 1B) and body weight loss was not negligible (Fig. S1B).

Combined, these results show that doublet therapies had a significant effect on lung cancer cells harboring EGFR T790M mutations; however, there is room for improvement in terms of the magnitude of remission and toxicity.

### 3.2 The efficacy and safety of mono‐ and combination therapies with a modified dose of afatinib, cetuximab, or bevacizumab

To develop the triplet therapy, we first assessed the efficacy and safety of modified doses of the monotherapy of each drugs. A modified dose of afatinib (10 mg·kg^−1^, 5 days per week, *n* = 6), cetuximab (0.1 mg per mouse, once a week, *n* = 6), or bevacizumab (2 mg·kg^−1^, twice a week, *n* = 6) was administrated to the RPC‐9 xenograft model mice for 4 weeks. Each monotherapy had only a slight effect on cell growth (Fig. S2A). Body weight loss was not observed in any treatment group (Fig. S2B). These results indicate that the modified monotherapy doses were tolerable, but that the effect of these therapies was very limited.

We next tested the combination therapies using the modified drug doses. Afatinib (10 mg·kg^−1^, 5 days per week) plus cetuximab (0.1 mg per mouse, once a week; *n* = 8), afatinib (10 mg·kg^−1^, 5 days per week) plus bevacizumab (2 mg·kg^−1^, twice a week; *n* = 8), or cetuximab (0.1 mg per mouse, once a week) plus bevacizumab (2 mg·kg^−1^, twice a week; *n* = 8) were administrated to mice harboring RPC‐9 xenograft tumors. No body weight loss was observed in either group (Fig. S2E,F), but the effect of the combination therapies was still limited (Fig. S2C,D). The tumors in each group were moderately inhibited during the treatment period; however, the tumors regrew during the observation period. Consequently, mono‐ or combo‐therapies using the modified drug doses were tolerable, but the effect was insufficient to inhibit lung cancer cells harboring EGFR T790M mutations.

### 3.3 Pathological complete remission was induced by triplet therapy with modified doses of afatinib, cetuximab, and bevacizumab in lung cancer cells harboring EGFR T790M mutations

**Figure 2:**
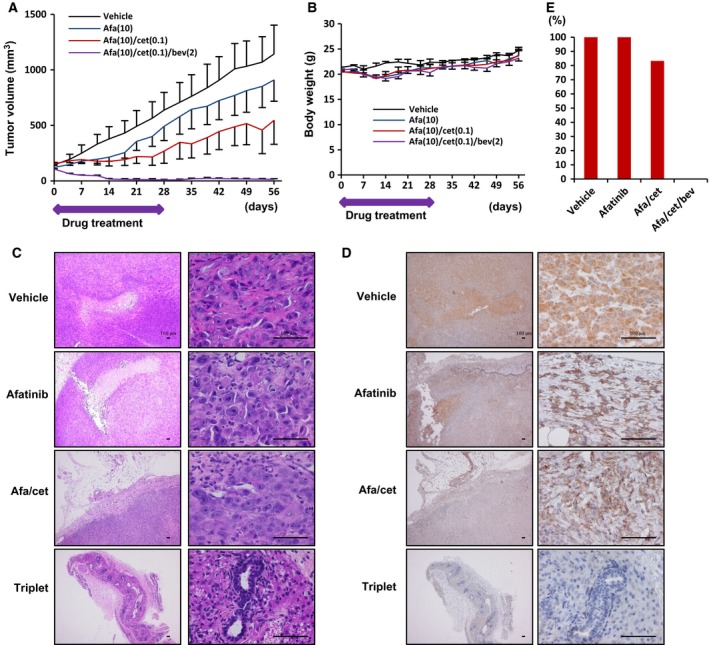
Deep remission induced by triplet therapy in xenograft tumors with RPC‐9 cells harboring EGFR exon 19Del + T790M mutations. Afa, afatinib; Cet, cetuximab; Bev, bevacizumab. (A) RPC‐9 cells were inoculated into nude mice and maintained when the tumor volumes reached 200 mm^3^. A modified dose of afatinib (10 mg·kg^−1^, five times per week p.o.), cetuximab (0.1 mg per body, once a week i.p.), or bevacizumab (2 mg·kg^−1^, twice a week i.p.) was given to each mouse (*n* = 6) for 4 weeks, followed by 4 weeks of drug cessation. Bars, SE. (B) Body weight loss was not observed in the mice. Bars, SE. (C) Microscopic examination of tumor tissues stained by hematoxylin and eosin. No cancer cells were detected in the tumors treated with the triplet therapy. (D) Immunohistochemistry examination revealed that cancer cells overexpressing EGFR were not observed in the tumors treated with the triplet therapy. Scale bar = 100 μm. (E) Cell line re‐establishment from xenograft tumors (*n* = 7). The enucleated tumors were minced and incubated *in vitro*. The tumors treated with triplet therapy could not be maintained *in vitro*.

To establish a more promising therapy, we sought to assess the efficacy and safety of triplet therapy using modified doses of afatinib (10 mg·kg^−1^, 5 days per week), cetuximab (0.1 mg per mouse, once a week), and bevacizumab (2 mg·kg^−1^, twice a week) *in vivo*. Surprisingly, the triplet therapy induced CR within the first 2 weeks in RPC‐9 xenograft tumors (Fig. 2A). Furthermore, the tumors treated with triplet therapy did not regrow during the four‐week observation period. Notably, no significant body weight loss was observed in any group (Fig. 2B).

**Figure 3:**
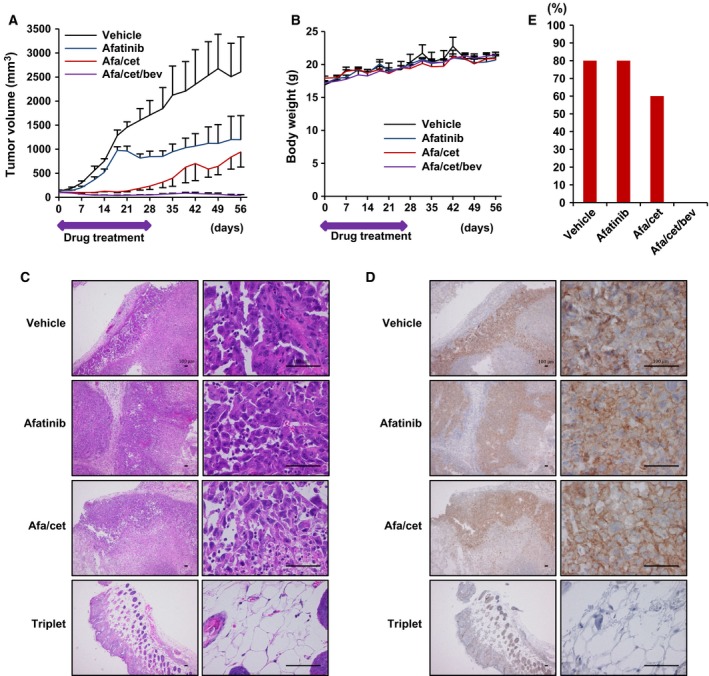
Deep remission induced by triplet therapy in xenograft tumors with H1975‐harboring L858R + T790M mutations. Afa, afatinib; Cet, cetuximab; Bev, bevacizumab. (A–E) The deep remission and safety induced by triplet therapy were re‐produced in a xenograft model with H1975 cells. Bars, SE. Scale bar = 100 μm.

To confirm the remission magnitude, tumors were enucleated 56 days after drug administration (i.e., 28 days after discontinuation of the treatment). The histology of these tumors was first assessed by a pathologist (T. Yoshino, Okayama University). As expected, no cancer cells were observed in the tumors treated with triplet therapy. In contrast, many cancer cells were observed in the tumors treated with vehicle, mono‐, or combination therapies (Fig. 2C). Total EGFR immunostaining was also examined as a tumor marker. No cells with EGFR overexpression were observed in tumors treated with triplet therapy, while many cancer cells with EGFR overexpression were found in the other groups (Fig. 2D). To test cancer cell viability in each of the tumors, we minced the tumors that were enucleated from the mice in culture medium (Fig. S3). Interestingly, no cancer cell lines could be re‐established from the tumors treated with triplet therapy. In contrast, almost all of the other tumors could be cultured in the same way as parental RPC‐9 cells (Figs 2E and S3). To confirm deep remission, the triplet therapy was tested in other lung cancer cell lines, namely H1975 cells harboring EGFR L858R and T790M mutations. Mice with H1975 cell xenograft tumors were treated with the same dose and schedule as mice with RPC‐9 tumors. The triplet therapy repeatedly induced deep remission in the H1975 cell xenograft tumors (Fig. 3A–E). Taken together, we conclude that the triplet therapy may induce deep remission in lung cancer cells with EGFR T790M mutations *in vivo*.

### 3.4 Mechanisms of deep remission induced by triplet therapy with modified dose of afatinib, cetuximab, and bevacizumab

**Figure 4:**
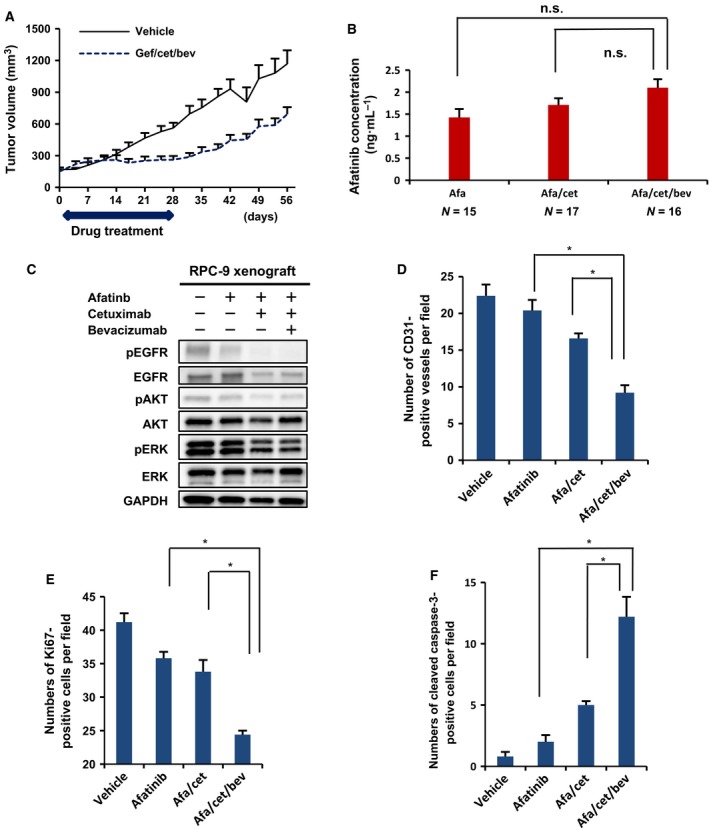
Mechanisms of the deep remission induced by triplet therapy. RPC‐9 cells were used in *in vitro* and *in vivo* models. (A) Triplet therapy with gefitinib, cetuximab, and bevacizumab did not induce deep remission in xenograft tumors. (B) The concentration of afatinib in the xenograft tumors was assessed by liquid chromatography–tandem mass spectrometry (LC‐MS/MS). Cetuximab and bevacizumab did not increase the concentration of afatinib in xenograft tumors. Bars, SE; n.s., not significant. (C–F) The xenograft tumors were treated for 1 week with the indicated drugs and collected for analysis. Afa, afatinib (10 mg·kg^−1^, five times per week p.o.); Cet, cetuximab (0.1 mg per body, once a week i.p.); or Bev, bevacizumab (2 mg·kg^−1^, twice a week i.p.). (C) The inhibitory effect on the EGFR signaling pathway in xenograft tumors was assessed by western blot. (D) The percent of CD31‐positive cells in the xenograft tumors treated with indicated drugs. Bars, SE. *P < 0.001. (E) The percent of Ki‐67‐positive cells in the xenograft tumors treated with indicated drugs. Bars, SE. *P < 0.001. (F) The percent of cleaved caspase‐3‐positive cells in the xenograft tumors treated with indicated drugs. Bars, SE. *P < 0.001.

We sought to investigate the mechanisms underlying the deep remission induced by the triplet therapy. First, to assess the role of afatinib, an alternative triplet therapy with ‘gefitinib’ instead of afatinib was administrated to mice with RPC‐9 xenograft tumors. The tumors were only moderately inhibited during the treatment period, and rapid tumor growth was observed during the observation period (Fig. 4A). Apparently, triplet therapy with gefitinib, cetuximab, and bevacizumab could not induce remission. This suggests that afatinib plays a crucial role in deep remission. Second, speculating that the drug concentration may play a role in the induction of deep remission, we measured the concentration of afatinib in the tumors treated with triplet therapy. Unexpectedly, the concentration of afatinib was not significantly increased in the tumors treated with triplet therapy compared with afatinib alone or afatinib plus cetuximab (Fig. 4B). Considering the results of this experiment, and the fact that intensive doses of afatinib plus cetuximab did not induce CR (Fig. 1A), we thought that the concentration of afatinib may not be crucial to achieve deep remission in our model.

To further investigate the mechanisms of deep remission *in vivo*, RPC‐9 xenograft tumors were collected after a week of treatment with afatinib alone, with afatinib and cetuximab, or with triplet therapy. The EGFR signaling pathway was assessed, and consistent with a previous report, phosphorylation of EGFR, AKT, and ERK was inhibited to a greater degree in tumors treated with afatinib and cetuximab compared to tumors treated with afatinib alone (Fig. 4C). However, the triplet therapy with afatinib, cetuximab, and bevacizumab did not have a superior inhibitory effect on EGFR signaling compared to afatinib plus cetuximab. This suggests that bevacizumab had little direct effect on the EGFR signaling pathway. Next, the expression of CD31 and Ki‐67 was examined through immunostaining to determine neovascularization and proliferation, respectively. As expected, CD31‐positive blood vessels were reduced significantly in tumors treated with triplet therapy compared to tumors treated with therapies without bevacizumab (Figs 4D and S4A). The number of Ki‐67‐positive cells was also smaller in tumors treated with triplet therapy compared to those treated with other therapies (Figs 4E and S4B). We then examined the expression of cleaved caspase‐3 by immunostaining to determine the level of apoptosis. The number of tumor cells with cleaved caspase‐3 expression was higher in the tumors treated with triplet therapy compared with the other groups (Figs 4F and S4C). Consistent with the result, the proapoptotic protein BIM was upregulated in the tumors treated with triplet therapy (Fig. S5). These results suggest that the greater suppression of neovascularization, the inhibitory effect on cell proliferation, and the induction of apoptosis by the triplet therapy may play important roles in the induction of deep remission *in vivo*.

## 4 Discussion

Resistance to EGFR‐TKIs is still a critical issue in EGFR‐mutant lung tumors. In this study, we discovered that triple therapy that included afatinib, cetuximab, and bevacizumab induced deep remission in xenograft tumors harboring EGFR T790M mutations. Earlier and deeper remission correlates with longer overall survival in Philadelphia chromosome‐positive chronic myeloid leukemia (Hughes *et al*., 2014). Complete and partial response could also be potential surrogate markers for predicting overall survival in lung cancer patients with EGFR mutations (Takeda *et al*., 2014). Therefore, the fact that we observed deep remission in response to afatinib, cetuximab, and bevacizumab *in vivo* is important.

Third‐generation EGFR‐TKI, osimertinib, has shown good inhibitory effect in resistant lung tumors harboring EGFR T790M mutations (Jänne *et al*., 2015). In this *in vivo* study, we confirmed the excellent inhibitory effect of osimertinib on xenograft tumors with RPC‐9 cells harboring EGFR T790M mutations (Fig. S6). The new compound almost completely inhibited tumor growth *in vivo*; however, most of the tumors regrew during the four‐week observation period post‐treatment. We also assessed the effect of combination therapy with osimertinib and bevacizumab. The effect of combination therapy was superior to that of osimertinib alone; however, most of the tumors regrew during the observation period (Fig. S6). This suggests that the inhibitory effect of osimertinib is excellent, but that the magnitude of remission has room for improvement. Finally, we also assessed the influence of preadministering osimertinib before triplet therapy treatment with afatinib, cetuximab, and bevacizumab in a xenograft model of RPC‐9 cells. Unfortunately, the effect induced by the triplet therapy seems to be attenuated in tumors pretreated with osimertinib for 2 weeks (Fig. S7). This suggests that the triplet therapy has difficulty overcoming osimertinib resistance in lung cancer harboring EGFR T790M. This might be explained by the studies that reported EGFR‐independent resistant mechanisms of osimertinib (e.g., MET amplification or RAS mutation; Kim *et al*., 2015; Ortiz‐Cuaran *et al*., 2016; Planchard *et al*., 2015). Alternative triplet therapy with osimertinib, cetuximab, and bevacizumab may be worth exploring.

The mechanisms of the deep remission induced by the triplet therapy may be complicated. The addition of cetuximab to afatinib (Regales *et al*., 2009) is one of the most important factors, as afatinib monotherapy (Miller *et al*., 2012), cetuximab monotherapy (Mukohara *et al*., 2005), or combinations of erlotinib and cetuximab (Janjigian *et al*., 2011), or gefitinib, cetuximab, and bevacizumab (Fig. 4A) had very limited effects in preclinical and clinical studies. In contrast, afatinib plus cetuximab produced a meaningful response (Janjigian *et al*., 2014; Regales *et al*., 2009). Weihua *et al*. (2008) showed that not only EGFR kinase activity but also kinase‐independent functions allow cancer cells to survive. Another recent preclinical study using an *in vitro* kinase assay showed that EGFR L858R + T790M preferentially dimerizes with wild‐type EGFR or ERBB2 on the cell surface (Red Brewer *et al*., 2013). In addition, Jia *et al*. (2016) showed the importance of EGFR dimerization inhibition using cetuximab for a mutant‐selective allosteric EGFR inhibitor, EA1045. These findings suggest why afatinib plus cetuximab have a greater effect in lung tumors harboring EGFR mutations (Regales *et al*., 2009). The reasons are as follows: (a) Afatinib can inhibit mutant EGFR and wild‐type EGFR and ERBB2 (Li *et al*., 2008) and (b) cetuximab is able to block wild‐type EGFR activation by interfering with ligand binding and dimerization (Li *et al*., 2005).

Several clinical trials have shown the significance of adding bevacizumab to EGFR‐TKIs (Herbst *et al*., 2011; Ichihara *et al*., 2015; Seto *et al*., 2014). One mechanism of this successful combination is the antiangiogenic effect. Another possible reason is the improved delivery of EGFR‐TKIs due to normalization of the tumor microenvironment (Chatterjee *et al*., 2014). However, in this study, the afatinib concentration was not increased in xenograft tumors. This is consistent with other *in vivo* preclinical experiments assessing the effect of bevacizumab on the erlotinib concentration (Li *et al*., 2014), and is also consistent with the finding that an increased dose of afatinib (25 mg·kg^−1^) plus cetuximab without bevacizumab did not induce a pathological CR in this study (Fig. 1A). A recent report suggested that the crosstalk between VEGFR and EGFR may be important for tumor growth; that report showed that dual malfunction of the EGFR and VEGFR genes resulted in complete tumor inhibition (Lichtenberger *et al*., 2010). Taken together, dual inhibition of the VEGFR and EGFR pathways may be one of the mechanisms underlying the induced deep remission seen in our model.

Our strategy was to use intensive dual blocking of driver oncoproteins with TKIs and antibodies combined with an antiangiogenic reagent. This strategy has already been used in clinical trials for solid tumors, including colon, breast, salivary gland, and lung cancers (Falchook *et al*., 2013a,b,c, 2014a,b). With the completion of these clinical studies, we expect that a trial using afatinib, cetuximab, and bevacizumab triplet therapy is clinically feasible. However, we would first need to consider the toxicity of this treatment, especially in terms of the skin rash and diarrhea that often result from wild‐type EGFR inhibition (Janjigian *et al*., 2014). Second, we have to consider the negative result of a clinical trial assessing the combination therapy with cytotoxic chemotherapy, cetuximab, and bevacizumab for colorectal cancer (Tol *et al*., 2009). However, we expect that dose modification could result in treatment tolerance while still having a sufficient effect.

In conclusion, we showed that triplet therapy with afatinib, cetuximab, and bevacizumab repeatedly induced pathological CR in lung cancers harboring EGFR T790M mutations with tolerable toxicity in preclinical xenograft models. The triplet therapy may have the potential to induce deep remission and prolong survival in patients with lung cancers harboring EGFR mutations. Clinical study of triplet therapy with afatinib, cetuximab, and bevacizumab is warranted.

## Conflict of interest

KO received a research grant from Novartis Pharmaceuticals, Japan. KH received honoraria from AstraZeneca, Eli Lilly Japan, Daiichi‐Sankyo Pharmaceutical, Boehringer‐Ingelheim, Nihon Kayaku, Taiho Pharmaceutical, Chugai Pharmaceutical, and Sanofi‐Aventis. KH also received research funding from Eli Lilly Japan, MSD, and Chugai Pharmaceuticals. KK received honoraria from Eli Lilly Japan, Nihon Kayaku, AstraZeneca, Daiichi‐Sankyo Pharmaceuticals, Chugai Pharmaceuticals, Taiho Pharmaceuticals, Boehringer‐Ingelheim, and Sanofi‐Aventis. All other authors declare no conflicts of interest regarding this study.

## Author contributions

KK, KO, and EI conceived and designed the experiments. KK, GM, HH, YT, and TY performed the experiments. KK, KO, GM, HH, YK, HK, YK, DM, TN, TK, EI, AS, KH, TY, MT, and KK analyzed and interpreted the data. KK and KO wrote and revised the manuscript. All authors read and approved the final manuscript.

## Supporting information


**Fig. S1.** Body weight loss in mice treated with afatinib plus cetuximab or afatinib plus bevacizumab.

**Fig. S2.** Efficacy and safety of monotherapy or combination therapies with modified doses of afatinib, cetuximab, and bevacizumab.

**Fig. S3.** Cell line re‐establishment.

**Fig. S4.** Mechanisms of the deep remission induced by triplet therapy.

**Fig. S5.** Expression of proapoptotic protein in xenograft tumors of PRC‐9 cells.

**Fig. S6.** Transient effect of osimertinib or osimertinib plus bevacizumab in xenograft tumors of RPC‐9 cells harboring EGFR exon 19Del + T790M mutations.

**Fig. S7.** Influence of preadministration of osimertinib in xenograft tumors of RPC‐9 cells harboring EGFR exon 19Del + T790M mutations.
