# Supplementary material for: Triplet therapy with afatinib, cetuximab, and bevacizumab induces deep remission in lung cancer cells harboring EGFR T790M in vivo
Source: Mol Oncol. 2017 May 2;11(6):670–81. doi: 10.1002/1878-0261.12063 (PMC5467494; doi:10.1002/1878-0261.12063)
Supplement: Supplementary file 1 — Fig. S1. Body weight loss in mice treated with afatinib plus cetuximab or afatinib plus bevacizumab. [file MOL2-11-670-s001.pptx]

## Slide 1
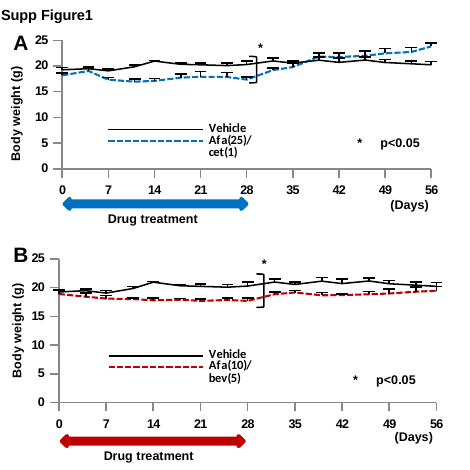

Supp Figure1
### Chart
| Category | Vehicle | Afa(25)/cet(1) |
|---|---|---|A
*
Body weight (g)
*　p<0.05
(Days)
Drug treatment
B
### Chart
| Category | Vehicle | Afa(10)/bev(5) |
|---|---|---|*
Body weight (g)
*　p<0.05
(Days)
Drug treatment
