# Supplementary material for: Triplet therapy with afatinib, cetuximab, and bevacizumab induces deep remission in lung cancer cells harboring EGFR T790M in vivo
Source: Mol Oncol. 2017 May 2;11(6):670–81. doi: 10.1002/1878-0261.12063 (PMC5467494; doi:10.1002/1878-0261.12063)
Supplement: Supplementary file 2 — Fig. S2. Efficacy and safety of monotherapy or combination therapies with modified doses of afatinib, cetuximab, and bevacizumab. [file MOL2-11-670-s002.pptx]

## Slide 1
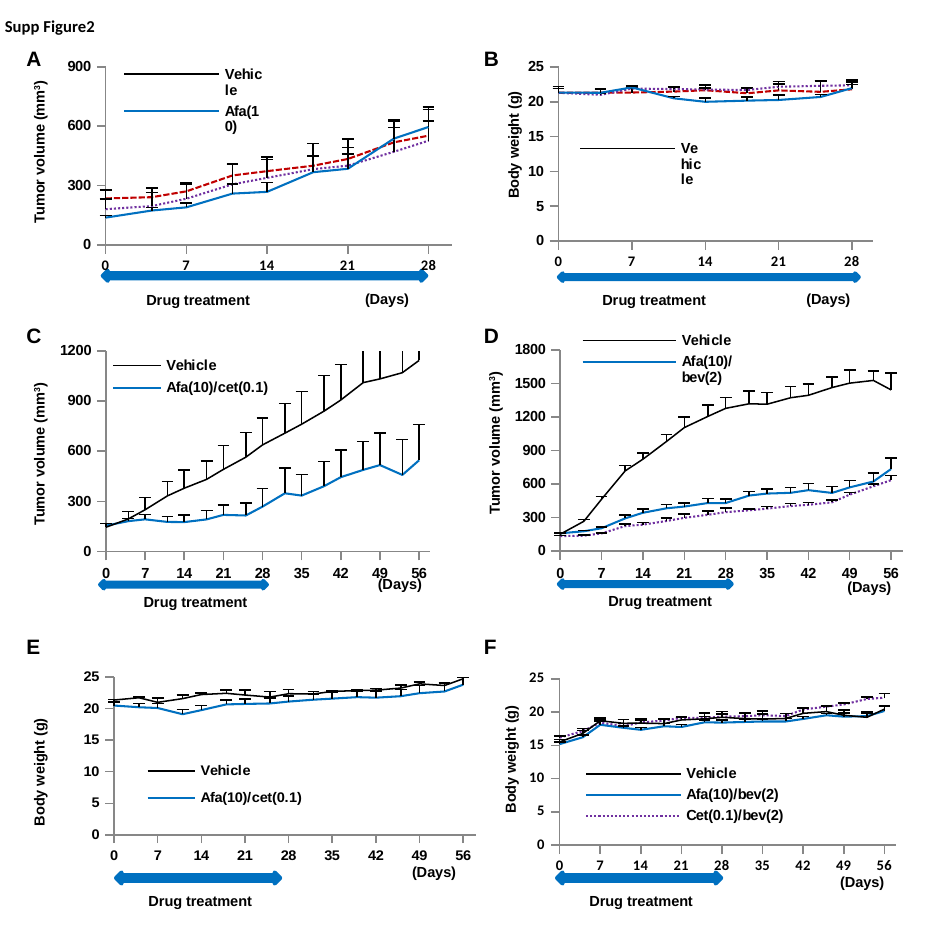

Supp Figure2
A
B
### Chart
| Category | Vehicle | Afa(10) | Cet(0.1) | Bev(2) |
|---|---|---|---|---|
### Chart
| Category | Vehicle | Afa(10) | Cet(0.1) | Bev(2) |
|---|---|---|---|---|Body weight (g)
Tumor volume (mm3)
(Days)
(Days)
Drug treatment
Drug treatment
C
D
### Chart
| Category | Vehicle | Afa(10)/bev(2) | Cet(0.1)/bev(2) |
|---|---|---|---|
### Chart
| Category | Vehicle | Afa(10)/cet(0.1) |
|---|---|---|Tumor volume (mm3)
Tumor volume (mm3)
(Days)
(Days)
Drug treatment
Drug treatment
E
F
### Chart
| Category | Vehicle | Afa(10)/bev(2) | Cet(0.1)/bev(2) |
|---|---|---|---|
### Chart
| Category | Vehicle | Afa(10)/cet(0.1) |
|---|---|---|Body weight (g)
Body weight (g)
(Days)
(Days)
Drug treatment
Drug treatment
