# Supplementary figures and images for: Triplet therapy with afatinib, cetuximab, and bevacizumab induces deep remission in lung cancer cells harboring EGFR T790M in vivo
Source: Mol Oncol. 2017 May 2;11(6):670–81. doi: 10.1002/1878-0261.12063 (PMC5467494; doi:10.1002/1878-0261.12063)

## Slide 1
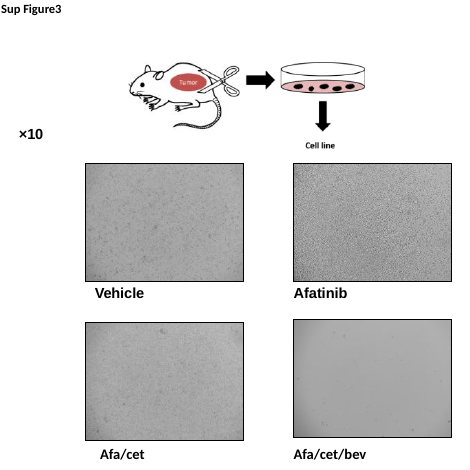

Sup Figure3
×10
Afatinib
Vehicle
Afa/cet
Afa/cet/bev

Supplement: Supplementary file 3 — Fig. S3. Cell line re‐establishment. [file MOL2-11-670-s003.pptx]

# Supp Figure 5

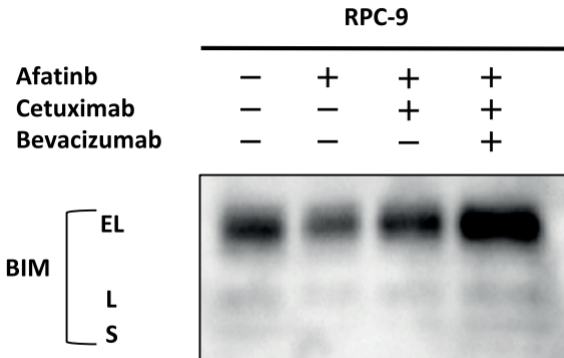

Supplement: Supplementary file 5 — Fig. S5. Expression of proapoptotic protein in xenograft tumors of PRC‐9 cells. [file MOL2-11-670-s005.pdf]
