# Supplementary material for: Triplet therapy with afatinib, cetuximab, and bevacizumab induces deep remission in lung cancer cells harboring EGFR T790M in vivo
Source: Mol Oncol. 2017 May 2;11(6):670–81. doi: 10.1002/1878-0261.12063 (PMC5467494; doi:10.1002/1878-0261.12063)

Supp. Figure 4A

CD31

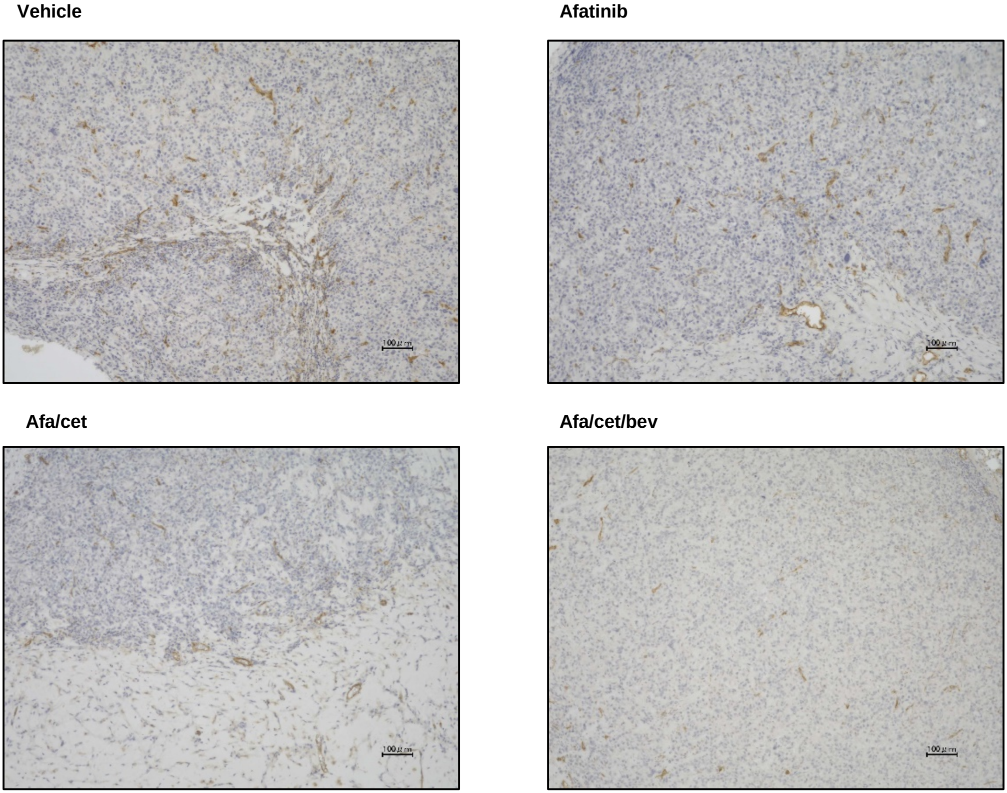

Supp. Figure 4B

Ki67

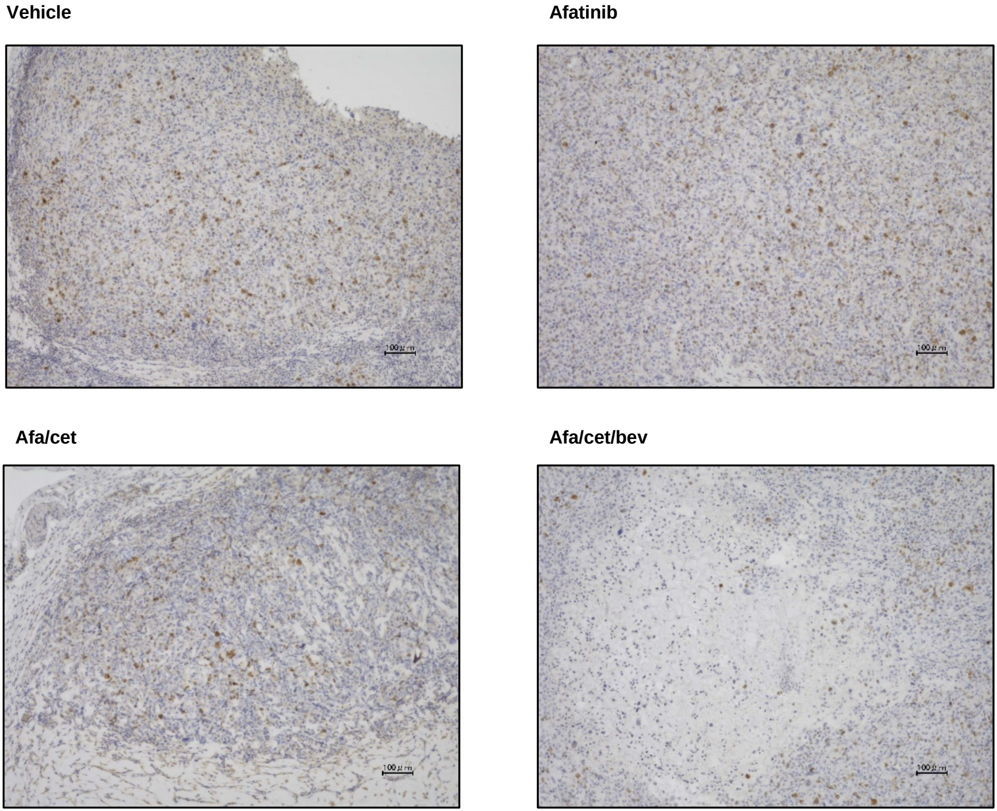

Supp. Figure 4C

Cleaved-caspase3

Vehicle

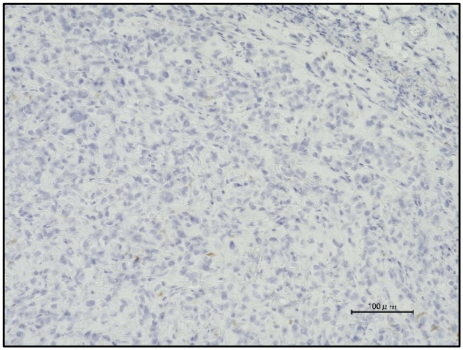

Afatinib

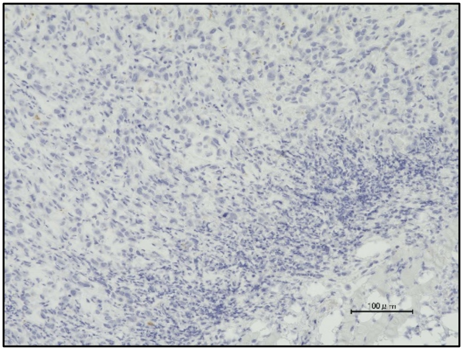

Afa/cet

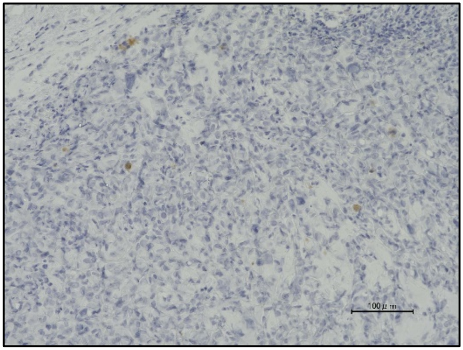

Afa/cet/bev

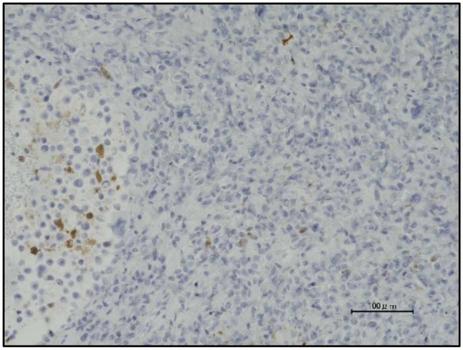

Supplement: Supplementary file 4 — Fig. S4. Mechanisms of the deep remission induced by triplet therapy. [file MOL2-11-670-s004.pdf]
