# Supplementary material for: Triplet therapy with afatinib, cetuximab, and bevacizumab induces deep remission in lung cancer cells harboring EGFR T790M in vivo
Source: Mol Oncol. 2017 May 2;11(6):670–81. doi: 10.1002/1878-0261.12063 (PMC5467494; doi:10.1002/1878-0261.12063)
Supplement: Supplementary file 7 — Fig. S7. Influence of preadministration of osimertinib in xenograft tumors of RPC‐9 cells harboring EGFR exon 19Del + T790M mutations. [file MOL2-11-670-s007.pptx]

## Slide 1
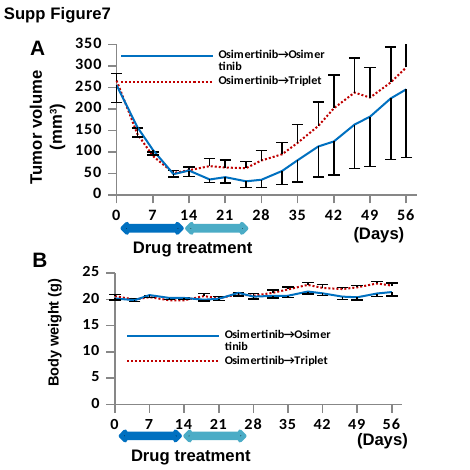

Supp Figure7
A
### Chart
| Category | Osimertinib→Osimertinib | Osimertinib→Triplet |
|---|---|---|Tumor volume (mm3)
(Days)
Drug treatment
B
### Chart
| Category | Osimertinib→Osimertinib | Osimertinib→Triplet |
|---|---|---|Body weight (g)
(Days)
Drug treatment
